# Postoperative glucocorticoids in patients with acute type A aortic dissection (GLAD): study protocol for a prospective, single-center, randomized controlled trial

**DOI:** 10.1186/s12871-023-02124-x

**Published:** 2023-05-15

**Authors:** Yi-zhi Deng, Ming-hao Luo, Jing-chao Luo, Jia-kun Li, Jia-qi Chen, Yi-jie Zhang, Jun-yi Hou, Ying Su, Guo-wei Tu, Zhe Luo

**Affiliations:** 1 Shanghai Medical College, Fudan University, Shanghai, 200032 China; 2 Cardiac Intensive Care Center, Zhongshan Hospital, Fudan University, Shanghai, 200032 China; 3 Department of Critical Care Medicine, Xuhui Central Hospital, Shanghai, 200032 China; 4 Shanghai Key Lab of Pulmonary Inflammation and Injury, Shanghai, China

**Keywords:** Acute type A aortic dissection, Organ dysfunction, Glucocorticoid, Methylprednisolone

## Abstract

**Background:**

Patients receiving surgical treatment of acute type A Aortic Dissection (aTAAD) are common to suffer organ dysfunction in the intensive care unit due to overwhelming inflammation. Previous studies have revealed that glucocorticoids may reduce complications in certain patient groups, but evidence between postoperative glucocorticoids administration and improvement in organ dysfunction after aTAAD surgery are lacking.

**Methods:**

This study will be an investigator-initiated, prospective, single-blind, randomized, single-center study. Subjects with confirmed diagnosis of aTAAD undergoing surgical treatment will be enrolled and 1:1 randomly assigned to receive either glucocorticoids or normal treatment. All patients in the glucocorticoids group will be given methylprednisolone intravenously for 3 days after enrollment. The primary endpoint will be the amplitude of variation of Sequential Organ Failure Assessment score on post-operative day 4 compared to baseline.

**Discussion:**

The trial will explore the rationale for postoperative application of glucocorticoids in patients after aTAAD surgery.

**Trial registration:**

This study has been registered on ClinicalTrials.gov (NCT04734418).

## Introduction

Acute type A aortic dissection (aTAAD) is a condition featuring high mortality, and surgery is the most common treatment. However, postoperative complications, such as multiple organ failure occur frequently and the prognosis remains to be improved [1].

One of the proposed mechanisms of postoperative complications is the disruption of immune homeostasis throughout the perioperative period. Surgical procedures may serve as a potential trigger for systemic inflammation [2]. Clinically, the occurrence of systemic inflammatory response syndrome (SIRS), which is believed to result in capillary leakage and other major postoperative complications, including ventricular dysfunction, respiratory failure, renal, hepatic and neurological dysfunction, bleeding, and ultimately, multiple organ failure [3], in the early postoperative period significantly increases the incidence of major adverse outcomes and mortality in patients with aTAAD [3, 4]. Inflammatory markers, such as interleukin(IL)-6, are good predictors of postoperative mortality [5]. Moreover, vascular paralysis induced distributive shock after major cardiovascular surgery is also associated with high mortality. At the core of its cause may be the activation of the inflammatory cascade with increased circulating levels of IL-1, IL-6, and tumor necrosis factor-α [6].

To alleviate the negative impacts of inflammatory response in the perioperative period of cardiac surgery, there have been successive studies reporting the effects of glucocorticoid (GC) administration in recent years. For instance, Dexamethasone for Cardiac Surgery (DECS) and Steroids in Cardiac Surgery trials have analyzed major clinical outcomes such as mortality after administrating GC during cardiac surgery [7, 8]. In addition, Halonen et al. revealed that the occurrence of postoperative atrial fibrillation was significantly lower after hydrocortisone administration as atrial fibrillation is believed to be bound up with postoperative inflammation [9, 10]. With regarding to vascular paralysis induced hypotension, recent clinical studies such as the APROCCHSS trial, have noted that the use of low doses of GC improves 90-day mortality and increases the likelihood of shock reversal in intractable shock [11].

However, numerous investigators have pointed out the use of GC in cardiac surgery remains controversial. Not only whether GC would lead to mortality benefits, but also how GC should be used are still inconclusive. Although meta-analysis has not revealed mortality benefits, certain patient groups may still benefit from GC [12]. More importantly, in previous clinical trials, GC was used prophylactically with a bolus style regimen, without sufficient tapering. Such a regimen may achieve inflammatory regulation at the expense of hypothalamic–pituitary–adrenal axis suppression.

The aim of this trial is to investigate whether postoperative GC, with a tapering regimen, would improve prognosis in patients with aTAAD undergoing surgery.

## Materials and methods

### Setting

This study will be an investigator-initiated, prospective, single-blind, randomized, single-center study, which will investigate the effect of postoperative GC on patients with aTAAD. The trial will be conducted in the cardiac intensive care center of Zhongshan Hospital, Fudan University.

### Overview

**Fig. 1:**
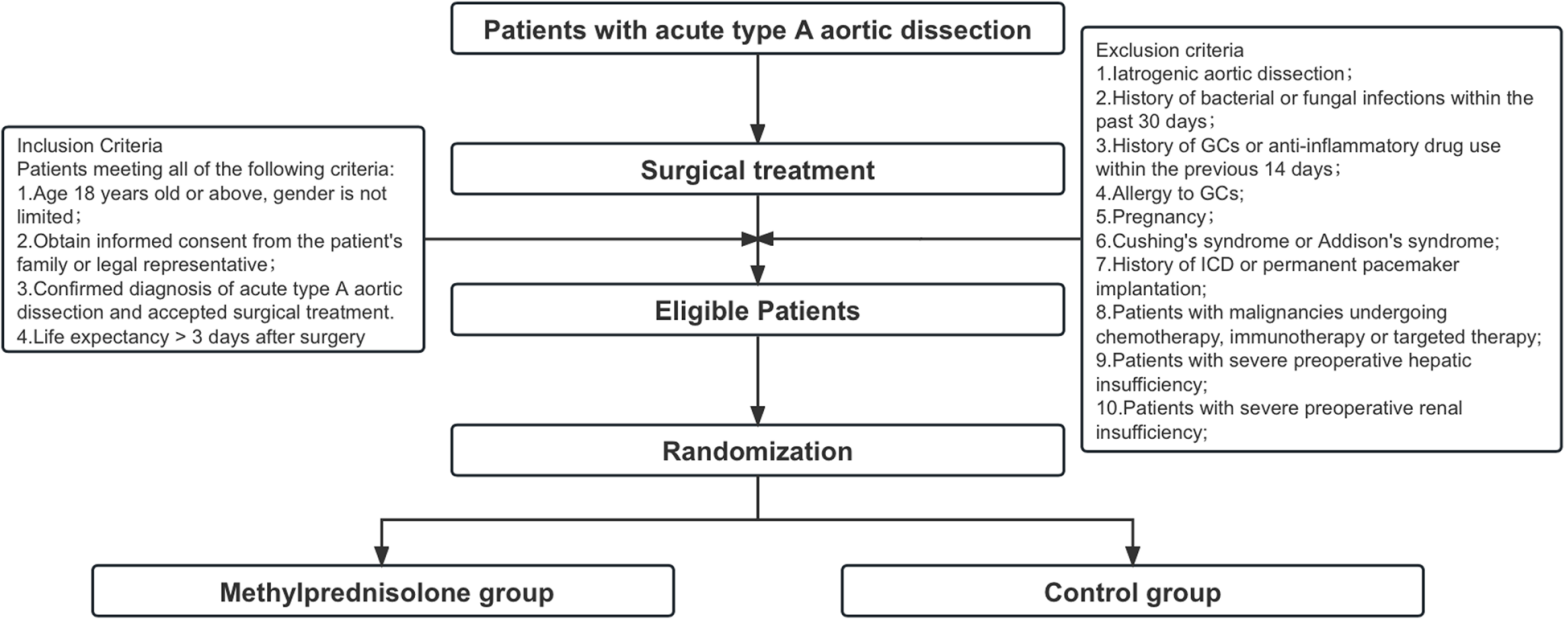
Flow chart of study protocol. GC:glucocorticoids; ICD: implantable cardioverter defibrillator

All patients with aTAAD undergoing surgery will be screened for study eligibility. Upon enrollment, eligible patients will be randomized 1:1 to one of the two treatment arms (i.e. GC or normal treatment) (Fig. 1). Data will be collected until hospital discharge or death.

### Study population

Inclusion and exclusion criteria are defined for enrolled patients;

#### Inclusion criteria

Patients meeting all of the following criteria:

- 1. Age 18 years old or above;
- 2. Obtain informed consent from the patient's family or legal representative;
- 3. Confirmed diagnosis of acute type A aortic dissection and received surgical treatment.
- 4. Life expectancy > 3 days after surgery.

#### Exclusion criteria

- 1. Iatrogenic aortic dissection;
- 2. History of bacterial or fungal infections within the past 30 days;
- 3. History of GCs or anti-inflammatory drug use within the previous 14 days;
- 4. Allergy to GCs;
- 5. Pregnancy;
- 6. Cushing's syndrome or Addison's syndrome;
- 7. History of implantable cardioverter defibrillator or permanent pacemaker implantation;
- 8. Patients with malignancies undergoing chemotherapy, immunotherapy or targeted therapy;
- 9. Patients with severe preoperative hepatic insufficiency;
- 10. Patients with severe preoperative renal insufficiency;

#### Criteria for withdrawing from the study

- 1. Allergy to GC during the study.
- 2. Complications that require surgical treatment.
- 3. Withdrawal of the informed consent.

### Intervention

**Fig. 2:**
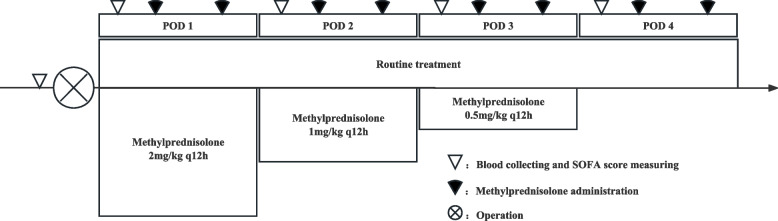
Diagram of interventions

All patients in the GC group will be given methylprednisolone (MP) intravenously for 3 days after entering the ICU postoperatively. MP will be injected every 12 h with a tapering dosage (Fig. 2).

The first dose of MP will be given on the morning of postoperative day (POD) 1 at a dosage of 2 mg/kg/d, followed by another dose with the same dosage 12 h later. On POD 2 and POD 3, MP will be reduced to 1 mg/kg/d and 0.5 mg/kg/d respectively, with 12 h intervals between two injections.

All patients in the control group will receive treatments as usual without the administration of GC. Patients will be required to be supervised by medical personnel when administering the study drug, and their vital signs will be checked regularly in case of emergency.

Strategies to improve adherence to intervention and monitor adherence to procedures will be implemented. Three dedicated research fellows will screen all patients who enter the ICU after surgery. They will be responsible to check if certain candidate meets the study criteria. They will also be in charge of ensuring that intervention will be prescribed based on the protocol. In addition, two independent intensive care specialists will monitor adherence to interventions throughout the study period.

### Outcomes

The primary outcome will be the difference of Sequential Organ Failure Assessment (SOFA) score on POD 4 compared to baseline (on POD1 before MP administration).

Secondary outcomes will be in-hospital mortality, days of ICU stay; days of hospital stay; duration of mechanical ventilation; tracheotomy; continuous renal replacement therapy; postoperative infection; inflammatory markers on POD 1, 2 and 3; composite endpoint (in-hospital morbidity and mortality or ICU stay over 30 days or tracheotomy).

### Safety outcomes

An adverse event is an incident that occurs after a patient or clinical trial subject receives a drug, but is not necessarily causally related to the treatment. All ICU staff will be instructed to pay attention to allergic reactions such as urticaria, shock and shortness of breath. These observations will be recorded on the participating patient’s data sheet and reported to investigators. Other extraordinary or questionable side effects will also be recorded.

Vital signs will be continually monitored and recorded in the ICU. The vital parameters include blood pressure, pulse, SpO_2_, temperature and respiratory rate. Blood counts, tests for infection, clinical chemistry biomarkers, and serum glucose will also be monitored. Prophylactic antibiotics will be used in all patients. If sepsis is suspected in the period when GC is given, treatment will follow the latest sepsis guidelines [13]. Electrolyte imbalance and hyperglycemia will also be corrected.

### Randomization and blinding

Block randomization will be conducted using a computer-generated random sequence, in blocks of size 4. Eligible patients will be assigned to one of the two study arms. A unique patient identification code will be generated to every screened patient without possible inference to patient identity.

Patients and researchers involved in data collection and analysis will be blinded. Doctors and nurses who will give the GC will not be blinded for allocations. These staff members will not be involved either in outcome evaluation or data analysis.

### ***Data to be collected*** (Table 1)

**Table 1:** Data to be collected

Time Points	Day of ICU admission	Treatment					
		Preoperative	Pre-drug	24 h	48 h	72 h	Discharge or death
Enrollment	x						
Informed consent	x						
Inclusion and exclusion criteria	x						
Demographics^a^	x						
Past medical history^b^	x						
Pre-operative cardiac function^c^	x						
Operative information^d^	x						
Assessment							
Temperature		x	x	x	x	x	
Respiratory rate		x	x	x	x	x	
Heart rate		x	x	x	x	x	
Systolic blood pressure		x	x	x	x	x	
Diastolic blood pressure		x	x	x	x	x	
SOFA score	x			x	x	x	
Methylprednisolone administration				x	x	x	
Inflammatory indicators^e^		x	x	x	x	x	
Blood count^f^		x	x	x	x	x	
Blood biochemistry^g^		x	x	x	x	x	
Arterial blood gas^h^		x	x	x	x	x	
Concomitant medications^i^		x	x	x	x	x	
Adverse events^j^				x	x	x	x
24 h fluid intake and output		x	x	x	x	x	

*TIA* Transient ischemic attack, *COPD* Chronic obstructive pulmonary disease, *DM* Diabetes mellitus, *NYHA* New York Heart Association, *LVEF* Left ventricular ejection fractions, *TAPSE* Tricuspid annular plane systolic excusion, *ECG* Electrocardiogram, *ALICE* Albumin-indocyanine green evaluation, *ICU* Intensive care unit, *CCB* Calcium channel blockers, *CRP* C-reactive protein, *PCT* Procalcitonin, *ESR* Erythrocyte sedimentation rate, *TNFα* Tumor necrosis factor-α, *IL-6* Interleukin-6, *IL-10* Interleukin-10, *pH* potential of Hydrogen, *CRRT* Continuous renal replacement therapy

^a^age, sex, height(cm), weight(kg), smoking and alcohol consumption

^b^pre-existing definite preoperative diagnosis; previous or screening period medical history (including at least hypertension, TIA, COPD, stroke, DM, various arrhythmias, peptic ulcer)

^c^preoperative NYHA classification, preoperative cardiac ultrasound including LVEF, TAPSE, preoperative ECG, myocardial enzyme profile, preoperative ALICE score for entrapment

^d^name of the procedure, duration of the procedure (min), time outside the body (min), time of block (min), time of transfer to the ICU. Intraoperative dose and time of hormone use, blood loss, and transfusion volume; β-blockers, CCB, anticoagulants such as aspirin, antiplatelet agents, antihypertensive agents, cardiotonic agents, proton pump inhibitors or H2-blockers, antibiotics used within the previous 7 days

^e^CRP, PCT, ESR, TNFα, IL-6, IL-10

^f^hemoglobin, white blood cell, neutrophil count, lymphocyte count, platelet count

^g^alanine aminotransferase, aspartate aminotransferase, total bilirubin, direct bilirubin, serum creatinine, plasma brain natriuretic peptide precursor, serum troponin T

^h^pH, partial pressure of oxygen, partial pressure of carbon dioxide, electrolytes (potassium ion, sodium ion), blood glucose

^i^β-blockers, CCB, anticoagulants such as aspirin, antiplatelet agents, antihypertensive drugs, cardiotonic drugs

^j^occurrence of various infections, CRRT, cerebrovascular events, gastrointestinal bleeding or perforation, cardiogenic shock, etc

Assessment of organ dysfunction: SOFA score.

Investigational medication: methylprednisolone.

Patient's inflammatory indicators (high sensitivity C-reactive protein, Procalcitonin, Tumor Necrosis Factor α, IL-6, IL-10).

Blood count: hemoglobin, white blood cell, neutrophil count, lymphocyte count, platelet count.

Blood biochemistry: alanine aminotransferase, aspartate aminotransferase, total bilirubin, direct bilirubin, serum creatinine, plasma brain natriuretic peptide precursor, serum troponin T.

Blood gas analysis: PH, partial pressure of oxygen, partial pressure of carbon dioxide, electrolytes (potassium ion, sodium ion), blood glucose.

Concomitant medications: β-blockers, calcium channel blockers, anticoagulants, antiplatelet agents, antihypertensive agents, cardiotonic agents, proton pump inhibitors or H2-blockers, antibiotics used within the previous 7 days.

Adverse events: occurrence of infections, gastrointestinal bleeding or perforation, etc.

Vital signs: including blood pressure, pulse rate, respiratory rate, body temperature.

### Data collection and storage

Blinded trial investigators will enter the baseline characteristics, process variables, and outcome data from the patient files into web-based case report forms (Electronic Data Capture System) from POD 1 to POD 4, and follow up at discharge from hospital. Calculation of the SOFA score will be done by the on-duty physician. Personal information about potential and enrolled participants will be collected and maintained. The initial of patient’s full name will be used instead of the real name to identify certain patients. In addition, during the study period, a unique identification number will be used as the dominant way to follow certain patients to protect confidentiality. If necessary, information stored in the electronic patient record will be used for follow-up and patients or their surrogates will be contacted directly if additional data is needed. Data will be stored in a third-party system and the trial investigators have full access to it.

### Clinical definitions

#### Inclusion criteria

##### Acute type A aortic dissection

Referring to patients with clinical manifestations such as chest pain, abnormal electrocardiogram or blood pressure, and confirmed by computerized tomography angiography.

#### Exclusion criteria

##### Iatrogenic aortic dissection

Referring to aortic dissection occurs in cardiac surgery intraoperatively or postoperatively with definite evidence.

##### Bacterial or fungal infections

Presence of clinical manifestations of systemic or local inflammatory response such as fever and elevated inflammatory indicators, and with or without support of bacterial or fungal pathogenic tests.

##### Cushing's syndrome

Referring to patients meeting the diagnostic criteria in clinical practice guideline of Cushing’s Syndrome 2021 [14].

##### Addison's syndrome

Referring to patients meeting the diagnostic criteria of Addison's syndrome [15].

##### Severe preoperative hepatic insufficiency

Referring to patients obtained 10–15 points in the Child-Turcotte-Pugh score.

##### Severe preoperative renal insufficiency

Referring to patients having acute or chronic kidney injury before surgery and meeting the indications for renal replacement therapy.

#### Outcomes

##### Postoperative infection

Referring to infection after cardiac surgery, including deep incisional surgical site infection occurring at the primary chest incision, deep incisional surgical site infection occurring at a secondary incision site, mediastinitis, infectious myocarditis or pericarditis, endocarditis, cardiac device infection, pneumonia, empyema, Clostridium difficile colitis, and bloodstream infection, with or without support of pathogenic evidence [16].

## Statistics

### Sample size

In this study, the SOFA score will be monitored continuously for 4 days after surgery. Our primary hypothesis is that the reduction in SOFA score of patients who will be treated with GC postoperatively would be greater than that in those treated with normal care. Based on our previous study, a decline of 2.87 ± 1.69 between the baseline SOFA score on POD1 and the one on POD 3 was observed in our patients [17]. Assuming a 25% increase in the reduction of the SOFA score in the GC group, a test level of α = 0.1 (bilateral), and a test efficacy of (1-β) = 0.9, it is expected that 96 cases are needed in each group, for a total of 192 cases. An estimate of a 10% dropout rate in these groups indicates that a sample size of 212 cases will be required.

### Data analysis

This study will summarize patient disposition, demographic and other baseline characteristics, study drug exposure, safety and efficacy. Statistical analyses will be performed using R, version 3.5.0 (R Foundation for Statistical Computing, Vienna, Austria). Continuous variables will be summarized descriptively based on mean (Mean), standard deviation (SD), median (Median) and maximum (Max) and minimum (Min) values, and comparisons of differences between groups will be based on whether the data meet a normal distribution with a two independent samples t-test or wilcoxon nonparametric test. The categorical variables will be summarized descriptively based on the number of subjects in each category and their corresponding percentage share using the chi-square test, and the Fisher's exact test will be used to analyze the differences between groups if necessary. Kaplan–Meier curves and log-rank test will be used to estimate hospital survival in patients in both arms.

In addition, intention to treat analyses which will include all participants randomly assigned to the study arms regardless of their loss to follow-up or compliance will be implemented. Per-protocol analyses, which include all participants with complete data sets, will also be performed for primary and secondary outcomes. In case of missing data, multiple imputation will be calculated.

An interim analysis will be conducted when the set reaches 50% of the proposed number of patients; if the primary endpoint has been reached and differences exist, the data safety monitoring board will discuss and vote on whether to allow early termination of the study. Unless otherwise specified, α = 0.05 (two-sided test) and *p* < 0.05 was defined as statistically significant.

### Subgroup analyses

The effect of the treatment among different subgroups: (1) gender, (2) diabetes, (3) The European System for Cardiac Operative Risk Evaluation (EuroSCORE) II, (4) Cardiopulmonary Bypass (CPB) duration, (5) age, (6) high sensitivity C-reactive protein on POD 1 and (7) SOFA on POD 1 will be analyzed. Stratified analysis using a logistic regression or Cox proportional hazards model, as applicable, will be performed. By adding a product term to the model that already contains the treatment and the subgroup factor, the interaction between each subgroup factor and the treatment group will be tested.

## Discussion

In this prospective, single-center, randomized controlled trial, we will investigate whether GC, with a tapering dosage, could improve organ dysfunction measured by the SOFA score in patients with aTAAD undergoing surgery.

GC is commonly used in cardiac surgery with much controversy [18]. As abundant evidence has shown that patients with aTAAD developing SIRS are more likely to suffer negative consequences, looking for novel effective treatments in this patient population to regulate such a pathophysiological process may be of paramount importance [19]. Previously, randomized controlled trials (RCTs) have emphasized on the clinical impact of intraoperative GC without considering postoperative inflammation control in cardiac surgery. Instead, in our study, sequential usage of GC will be achieved at a dosage that is widely used to treat acute severe inflammation-related conditions. Although the duration is relatively short (3 days), it fits in the clinical context and guarantees the feasibility of the study because some stable patients may leave ICU in 3 days. In addition, choosing indicators (SOFA Score) reflecting organ dysfunction increases the clinical value of the research. Data from the International Registry of Aortic Dissection has shown that organ failure is one of the leading causes of death in patients with aTAAD [1]. Examining the influence of GC with respect to the SOFA score could address this clinical quandary and potentially enhance patient survival rates.

There are a variety of strengths in our study. 1) The effect of GC in patients with aTAAD undergoing surgery is lacking, especially for those undergoing total aortic arch replacement with frozen elephant trunk implantation. Such a patient population is greatly exposed to the risk of SIRS and subsequent deterioration in organ function. This study will be the first RCT to investigate the role GC could play in these patients. 2) Former interventions of GC in cardiac surgery generally adopted a pulse regimen without tapering [7, 20]. The dosage of GC was particularly high (eg. 1 g MP) when given prophylactically before the initiation of CPB. However, benefits of GC to regulate dysregulated inflammation have mostly been observed in critical care studies adopting a mild-to-moderate dose of GC, as we have seen in inflammation-mediated conditions, such as acute respiratory distress syndrome [21–23]. In our study, we will implement an initial dosage of 4 mg/kg/d MP on POD 1 and taper it to 1 mg/kg/d on POD 3. Such a regimen will provide novel evidence on whether sequential and tapered use of GC would be better for patient prognosis. 3) A reduction in the SOFA is a reliable indicator of improvement in organ function and survival potential. Organ failure is one of the most prominent causes of death in surgical patients of aTAAD based on the data from international registry. Unfortunately, accurate definition of organ dysfunction is relatively missing, especially when multiple organs are involved. Previously, we have demonstrated that the SOFA score is associated with and could be used as a good predictor for all-cause in-hospital mortality and a lower SOFA score is linked to a decreased possibility of death after surgery, especially the one on POD 2 and POD 3 [17]. As our primary outcome would look at the difference between the SOFA score on POD 1 and POD 4, we believe such a choice could further elucidate the clinical value of the SOFA score. 4) Multiple biomarkers, such as CRP and IL-6, will be recorded in the research, which has the potential not only to depict a more accurate picture of inflammatory state in patients with aTAAD, but also to monitor and compare the effect of GC on inflammation as part of our secondary outcomes.

At the same time, it should be noted that several limitations may exist. First, as a single-center study, variations between different centers and clinical practice will not be considered, which may restrict the validity of the result in certain clinical and social context. Secondly, intraoperative GC will not be included as part of the research protocol as our current trial members work independently from the anesthesia team. As a result, the majority of patients will receive GC before the initiation of CPB even if they will be randomized to the control group in this study. However, it will still be comparable as patients in the control group will be free from sequential and tapering usage of GC postoperatively.

## Data Availability

Data sharing is not applicable to this article as no datasets were generated or analysed for this study protocol.

## Abbreviations

aTAAD: Acute Type A aortic dissection
SIRS: Severe inflammatory response syndrome
IL: Interleukin
GC: glucocorticoid
DECS: Dexamethasone for Cardiac Surgery
GLAD: Glucocorticoids in Patients with acute type A Aortic Dissection
ICU: Intensive care unit
POD: Postoperative day
MP: Methylprednisolone
SOFA: Sequential organ failure assessment
CPB: Cardiopulmonary bypass
CRP: C-reactive protein
